# Host-Related Laboratory Parameters for Leprosy Reactions

**DOI:** 10.3389/fmed.2021.694376

**Published:** 2021-10-22

**Authors:** Yuqian Luo, Mitsuo Kiriya, Kazunari Tanigawa, Akira Kawashima, Yasuhiro Nakamura, Norihisa Ishii, Koichi Suzuki

**Affiliations:** ^1^Department of Laboratory Medicine, Nanjing Drum Tower Hospital and Jiangsu Key Laboratory for Molecular Medicine, Nanjing University Medical School, Nanjing, China; ^2^Department of Clinical Laboratory Science, Faculty of Medical Technology, Teikyo University, Tokyo, Japan; ^3^Department of Molecular Pharmaceutics, Faculty of Pharma-Science, Teikyo University, Tokyo, Japan; ^4^National Sanatorium Tamazenshoen, Tokyo, Japan

**Keywords:** leprosy, leprosy reactions, T1R, ENL, biomarkers, correlates

## Abstract

Leprosy reactions are acute inflammatory episodes that complicate the course of a *Mycobacterium leprae* infection and are the major cause of leprosy-associated pathology. Two types of leprosy reactions with relatively distinct pathogenesis and clinical features can occur: type 1 reaction, also known as reversal reaction, and type 2 reaction, also known as erythema nodosum leprosum. These acute nerve-destructive immune exacerbations often cause irreversible disabilities and deformities, especially when diagnosis is delayed. However, there is no diagnostic test to detect or predict leprosy reactions before the onset of clinical symptoms. Identification of biomarkers for leprosy reactions, which impede the development of symptoms or correlate with early-onset, will allow precise diagnosis and timely interventions to greatly improve the patients' quality of life. Here, we review the progress of research aimed at identifying biomarkers for leprosy reactions, including its correlation with not only immunity but also genetics, transcripts, and metabolites, providing an understanding of the immune dysfunction and inflammation that underly the pathogenesis of leprosy reactions. Nevertheless, no biomarkers that can reliably predict the subsequent occurrence of leprosy reactions from non-reactional patients and distinguish type I reaction from type II have yet been found.

## Introduction

### Leprosy

Leprosy, also known as Hansen's disease, is an age-old disease, and patients with leprosy have been ostracized by their communities and families throughout history (1–3). Leprosy is an infectious disease caused by *Mycobacterium leprae* (*M. leprae*), an acid-fast, rod-shaped bacillus that preferentially infects macrophages (histiocytes) in the dermis and Schwann cells (SCs) in peripheral nerves (4). Thus, the disease mainly affects the skin and the peripheral nerves; however, mucosa of the upper respiratory tract and eyes are also affected (5). Nerve damage may result in a lack of ability to feel pain, potentially leading to the loss of extremities from repeated injuries or infection due to unnoticed wounds. Therefore, leprosy is the second most severe human mycobacterial disease after tuberculosis (6). Nerve damage occurs due to direct invasion of SCs by *M. leprae* and the subsequent host immune response resulting in inflammation; however, the precise mechanism is still unclear (6). *Mycobacterium lepromatosis* (*M. lepromatosis*) is a comparatively new bacterium which causes severe form of leprosy, namely diffuse lepromatous leprosy (DLL), through nerve invasion and extensive skin ulcerations (7).

The clinical manifestations of leprosy depend on the magnitude of the host immune response to *M. leprae*, and can be classified based on decreasing immune responses as tuberculoid (TT), borderline tuberculoid (BT), mid-borderline (BB), borderline lepromatous (BL), and lepromatous (LL) (8). LL is characterized by low or limited cell-mediated immune responses to *M. leprae*, with a lack of *M. leprae*-specific T cells, increased regulatory T cells, and high levels of *M. leprae*-specific antibodies, allowing the proliferation of *M. leprae* within and around macrophages (8). In contrast, TT features a vigorous pro-inflammatory Th1 and Th17 immune response, leading to elimination or containment of *M. leprae* in granulomas and collateral damage of the host cells, mimicking autoimmunity (8, 9). The majority of patients are classified as the three borderline types, BT, BB, and BL, which exhibit a relatively unstable immunological state (8). Comparatively simpler methods for classification of leprosy include paucibacillary and multibacillary forms. Literally, paucibacillary patients are those with a small number of skin lesions (<5 skin lesions) and a low bacillary load, whereas multibacillary patients are those with numerous infiltrated skin lesions (>5 skin lesions) displaying high bacillary loads (10). In 1980s, the WHO recommended a 6-month multidrug therapy (MDT) for paucibacillary and a 12-month MDT for multibacillary cases (https://www.who.int/lep/resources/9789290226383/en/).

### Leprosy Reactions

Leprosy reactions (LRs) are acute nerve-destructive inflammatory episodes that complicate the course of *M. leprae* infection and are the major cause of leprosy-associated disabilities. Currently, there is no diagnostic test to detect or predict LRs before the onset of clinical symptoms. Similar inflammatory reactions (called paradoxical reactions) occur in other mycobacterial diseases, such as tuberculosis and *Mycobacterium ulcerans* infection (Buruli ulcer) during the natural course of infection or following antibiotic treatment (11, 12). However, the incidence and severity of these reactions are much higher in leprosy (13–16). LRs may occur before, during, or even after the successful completion of MDT, and up to 50% of leprosy patients experience at least one LR during their lifetime (15, 16). The timing of LRs has implications for the clinical diagnosis, adherence to MDT, and differentiation of relapse or re-infection. Immunomodulatory drugs, such as steroids, are required to treat LRs, and high doses are often required over prolonged periods, potentially contributing to morbidity (15, 16).

Two types of LRs with relatively distinct clinical and pathological features can occur: type 1 reaction (T1R; also known as reversal reaction) and type 2 reaction (also known as erythema nodosum leprosum; ENL). T1R is characterized by acute inflammation in pre-existing leprosy lesions in the skin and peripheral nerves, resulting in edema, which is sometimes accompanied by ulcerative lesions (16, 17). Although edema of the hands, feet and face can also be a feature of LRs, systemic symptoms are unusual (16, 17). Involvement of the peripheral nerves leads to a loss of function of both sensory and motor nerves with tenderness and pain. A nerve abscess may rarely occur in T1R, causing swelling, tenderness and ultimately nerve impairment (16, 17). The diagnosis of T1R is usually made clinically, but a skin biopsy is sometimes performed to support the diagnosis. The histological features of T1R include edema with disorganization of the granuloma and widespread infiltration of inflammatory cells, consisting of lymphocytes, epithelioid cells, and giant cells (17). Once it lacked standardized tool for assessing reactions, now a reaction clinical severity scale has been used to measure clinical features and treatment outcomes (18).

In contrast to T1R, ENL is a systemic inflammatory response characterized by neutrophil infiltration, activation of the complement system, extravascular deposition of immune complex, and secretion of pro-inflammatory cytokines in both skin lesions and peripheral blood (15, 19, 20). The Erythema Nodosum Leprosum International STudy (ENLIST) Group has defined a severity scale for assessing ENL and collected data on its clinical features to improve evidence-based treatments for ENL (21, 22). Skin lesions of ENL often show a perivascular infiltrate of neutrophils in the dermis and subcutaneous tissues with erythematous lesions with tender papules or nodules, and may ulcerative and become necrotic often accompanied by fever and malaise (15, 23). Peripheral edema of the limbs and face is common in patients with ENL, and the number of neutrophils in a skin biopsy diminishes with the age of the lesion (15, 24).

ENL affects numerous organ systems and is a painful inflammatory complication of leprosy (25). Impairment of nerve function presents in over 50% of ENL (15, 23). Both large and small joints are frequently affected in ENL (23), and painful lymphadenopathy occurs in 15% of ENL cases (26). Testicular tenderness and severe inflammation occur in 13.5% of male ENL patients (23, 27). Nasal involvement occurs in 8% of ENL patients and may lead to septal perforation (28). In addition, there are occasional reports of pulmonary infiltrates associated with ENL (29). Ocular inflammation is also reported in 5% of cases, and ENL is associated with iridocyclitis, episcleritis, and scleritis (30). Furthermore, hemophagocytic syndrome (31), secondary amyloidosis (32), nephrotic syndrome, and glomerulonephritis (33) are also associated with ENL.

Borderline leprosy is immunologically unstable and more prone to developing LRs (8). T1R reflects a sudden shift toward Th1 immune responses and is most frequently associated with BT, BB, or BL, characterized by CD4+ T cell infiltration in skin and nerve lesions, resulting in nerve damage (34). ENL reactions primarily occur in patients with LL or BL with large bacterial burdens and reflect increased cell-mediated and humoral immune responses to *M. leprae* components (34). Although the precise mechanisms of the reactions-associated nerve damage are unclear, it may involve immune injury due to the release of inflammatory cytokines or activity of CD8+ cytotoxic T lymphocytes (CTLs), ischemia due to edema within the perineural sheath, apoptosis, and demyelination (6, 35, 36).

In addition to T1R and ENL, Lucio's reaction is a rare reactional state seen in patients with DLL, characterized by recurrent multiple and extensive areas of ulcerations affecting the extremities (37).

**Figure 1:**
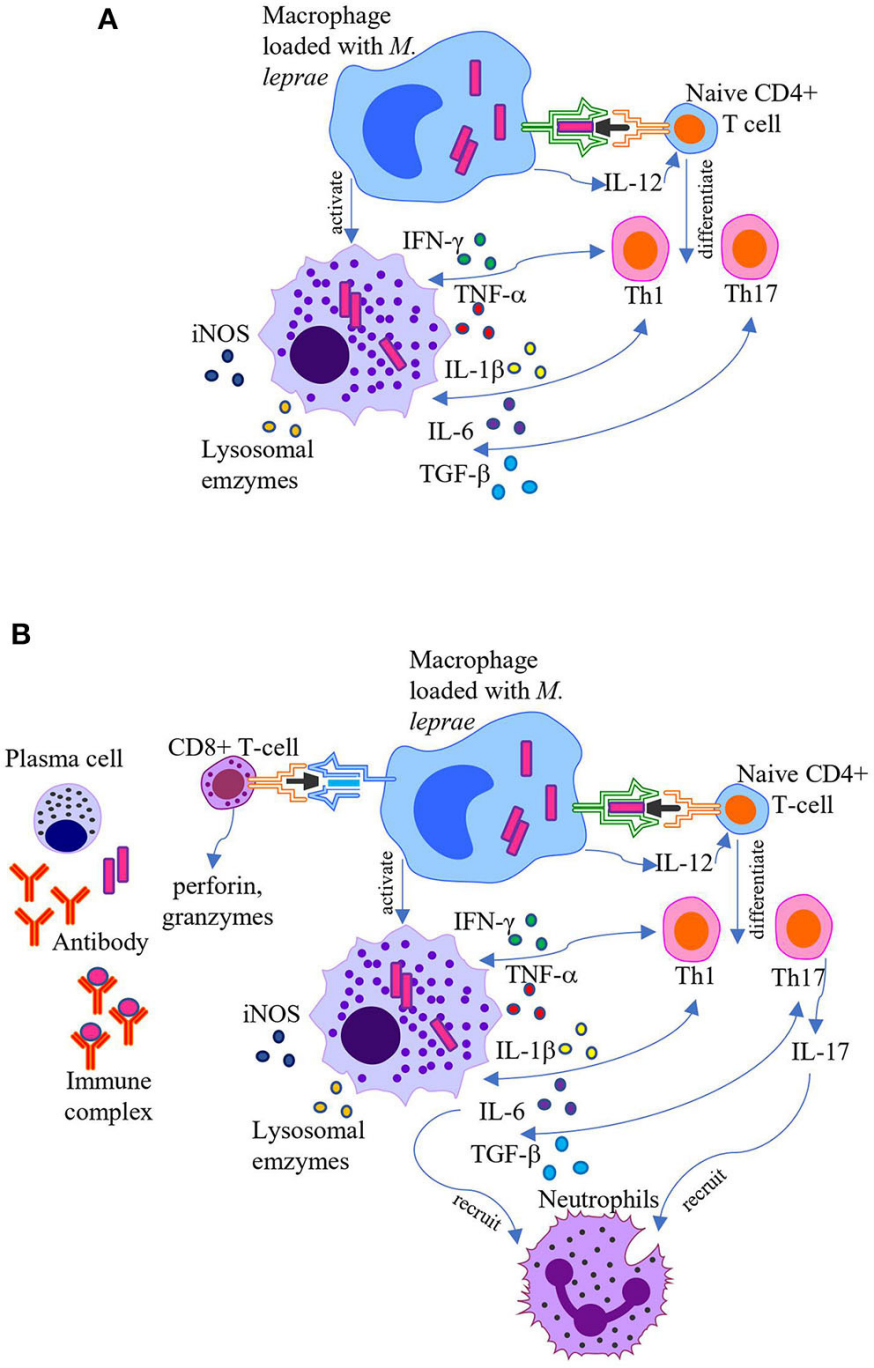
Pathogenesis of leprosy reactions. Schematic representation of the current understanding of pathogenesis of T1R and ENL. **(A)** T1R occurs due to overactive CD4+ T cell-mediated cellular immune responses. Activated macrophages release proinflammatory cytokines, such as TNF-α, IFN-γ, IL-1β, IL-6, lysosomal enzymes, and reactive oxygen species which cause leakiness in the endothelial barrier and tissue injury, enhancing immune cell migration into the area. **(B)** ENL is associated with formation of immune complexes, increased CD4+/CD8+ T cell subset ratio in both peripheral blood and skin, and recruitment of neutrophils. Activated macrophages in concert with neutrophils and T cells secret high levels of pro-inflammatory cytokines.

Neuropathy is often irreversible if the diagnosis and intervention of LRs are delayed beyond 6 months following symptoms (35). Therefore, identifying diagnostic and predictive biomarkers for LRs is essential, allowing for precise diagnosis and timely interventions to significantly improve patient prognosis and quality of life (QOL). As the clinical manifestations of leprosy are mirrored by the host immune response against *M. leprae*, leprosy is also considered a human immunoregulatory disease; thus, host immune-associated biomarkers have been extensively explored for their potential to correlate with and predict the disease state. Below, we review the progress of studies aiming to identify host-biomarkers for LRs, providing further understanding of LR pathogenesis (Figure 1). Literatures were handed search through PubMed (http://pubmed.ncbi.nim.nih.gov) from November 1, 2020, using keywords including leprosy reactions; reversal reactions; ENL; biomarkers. To be noted, out of numerous published studies of LRs, only a small part have provided scientifically accurate data (15). And no correlate seen in LRs yet fulfill the requirement of specificity and sensitivity for diagnosis biomarkers.

## Pathogenesis and Potential Biomarkers for T1R

### T Cell-Mediated Hypersensitivity

T1R is induced by T cell-mediated hypersensitive reactions that predominantly occur in the borderline forms of leprosy (38). Hypersensitivity indicates that the host immune system has responded to the pathogen or its derivatives in a way that ultimately damages the host, as opposed to protecting it. Both CD4+ helper T (Th) cells and CD8+ CTLs contribute to tissue damage in T1R (39, 40). The immune response to *M. leprae* is initiated following the colonization of the nasal mucosa, potentially of the nasal cavity, and subsequent phagocytosis by antigen-presenting cells (APCs), such as dendritic cells and macrophages. The APCs then migrate to the regional lymph nodes, where they present the antigen on its surface via major histocompatibility complex (MHC) class II molecules to naïve CD4+ Th cells.

CD4+ Th cell activation occurs following the binding of the T cell receptor (TCR) and the CD4 co-receptor to the antigen-MHC class II complex of APCs (signal one). In addition, CD28 on the surface of CD4+ Th cells binds to B7 molecules (CD80/CD86) on the surface of the APC (signal two). Once APCs bind to CD4+ Th cells, they release interleukin (IL)-12 (signal three), a cytokine that dictates naïve CD4+ Th cell differentiation into a mature type 1 Th cell (Th1). At this point, the CD4+ Th cell becomes an effector cell and can release the cytokine IL-2, which contributes to the proliferation of both CD4+ Th cells (autocrine) and other cells (paracrine). Interferon-γ (IFN-γ) is also secreted by APCs, inducing Th1 proliferation and macrophage activation (41). Activated macrophages release proinflammatory cytokines, such as tumor necrosis factor (TNF)-α, IL-1β, and IL-6, which cause leakiness in the endothelial barrier, enhancing immune cell migration into the area, all of which leads to local edema, redness, and warmth (41, 42). Activated macrophages also secrete lysosomal enzymes, complement components, and reactive oxygen species into the exposed area, all of which contribute to tissue injury (41, 42). Similarly, Th1 cells and T cell-mediated hypersensitivity contribute to damage of the myelin sheath around nerve fibers in multiple sclerosis (43) and intestinal mucosa inflammation in inflammatory bowel disease (IBD) (44).

Naïve CD4+ Th cells can also differentiate into Th17 cells in response to IL-6 and transforming growth factor (TGF)-β secreted by APCs (45–47). Following activation, Th17 cells produce and secrete IL-17, which facilitates neutrophil recruitment (45). Furthermore, CD8+ CTLs contribute to tissue damage via direct cytotoxicity (39, 40). CD8+ CTLs recognize antigens bound to MHC class I molecules, present on all nucleated cells of the body (48). Following antigen recognition via the TCR, effector CD8+ CTLs release perforin and granzymes from intracellular granules (48). Perforin perforates the target cell membrane to form pores, allowing granzymes to enter the cell and induce apoptosis (48).

### Antigen Responsiveness

As described above, T1R occurs due to overactive T cell-mediated cellular immune responses upon recognition and presentation of *M. leprae* antigens by APCs. APCs recognize *M. leprae* molecules via pattern recognition receptors (PRRs), such as toll-like receptors (TLRs) (49). In leprosy, cell surface heterodimers of TLR1/TLR2 and TLR6 recognize molecular patterns of *M. leprae*, such as peptidoglycan (PGN) and lipoarabinomannan (LAM), mediating APC activation (50–52). In leprosy lesions, TLR2 was shown to mediate SC apoptosis, contributing to nerve injury characteristic of T1R (53). Furthermore, a study of 21 Nepalese patients demonstrated that TLR2 and TLR4 expression is associated with T1R, and corticosteroid treatment reduced gene and protein expression of TLR2 and TLR4 (54).

Consistent with a role of TLRs in T1R, polymorphisms in TLR genes influence the risk of acquiring leprosy and developing T1R, putatively due to their role in APC responsiveness to *M. leprae*. In a cohort of Ethiopian patients, a single nucleotide polymorphism (SNP) in TLR2 (597C > T) was associated with protection against T1R, while a 280-bp microsatellite marker was associated with an increased risk of T1R (55). In addition, the TLR4 SNP (1530G > T) is more frequently seen in individuals with T1R (56). A cohort of 238 Nepalese patients found that the non-synonymous polymorphism rs5743618 of TLR1 (I602S) was protective against T1R (57). The I602S SNP of TLR1 inhibits surface trafficking of the TLR1/TLR2 dimer, resulting in hypo-responsiveness to mycobacteria, suggesting a potential protective mechanism against *M. leprae* (58, 59). In addition, the MHC gene region carries major susceptibility for leprosy and LRs in different populations, with both protective and risk alleles (60, 61).

### Pro-inflammatory Cytokines

Compared with non-reactional patients, enhanced Th1 responses and macrophage activation in T1R is demonstrated by a pro-inflammatory Th1 cytokine profile, including IFN-γ, TNF-α, IL-1β, IL-6, IL-2, soluble IL-2 receptors, IL-12, TGF-β, and inducible nitric oxide synthase (iNOS), in the blood, skin, and nerves (62–64). Therefore, these cytokines are potential predictive biomarkers for LRs. Monitoring serum cytokines in newly diagnosed leprosy cases before starting therapy and during reactional episodes indicates that elevated serum TNF-α, IFN-γ, and IL-1β levels predict T1R development (65, 66). An analysis of 27 plasma factors also revealed increased plasma IL-6 levels in both T1R and ENL compared to patients with non-reactional leprosy (67). IL-6 promotes cell-mediated immune reactions by stimulating IL-17 production and inhibiting regulatory T cells (Tregs) (68).

#### TNF-α

The pro-inflammatory cytokine TNF-α belonging to the TNF superfamily (TNFSF) is increased in the skin, serum, and nerves during T1R (69–71). Genome-wide association studies (GWAS) showed that SNPs in the *TNFSF15-TNFSF8* locus are associated with excessive inflammatory responses in T1R (72), but effects may vary with age (73). GWAS also identified T1R-specific associations with variants of leucine rich-repeat kinase 2 (*LRRK2*), which may cause pro-inflammatory responses (74). Interestingly, peripheral nerve damage due to inflammation in T1R and neuroinflammation in Parkinson's disease share overlapping genetic control of pathogenicity (75).

#### IFN-γ, iNOS

In T1R lesions, IL-12 is consistently expressed, IL-4 is absent, and IFN-γ producing CD4+ Th cells and CD8+ CTLs are selectively increased during *M. leprae* clearance and concomitant tissue damage (39, 40). Th1 cell activation and IFN-γ production are critical for an efficient immune response against *M. leprae* (42). IFN-γ enhances *M. leprae* antigen presentation by increasing MHC and co-stimulatory molecule expression and activates the antimicrobial response (76). IFN-γ is crucial for macrophage plasticity, as it polarizes naïve M0 macrophage to M1 pro-inflammatory macrophages, which produce cytokines and iNOS (41). iNOS generates reactive nitrogen radicals involved in mycobacteria killing (41), and high levels of iNOS are identified in skin biopsies from T1R lesions (63). Another study found that the macrophage activation marker, neopterin, is a useful biomarker in monitoring T1R patients during corticosteroid therapy (77).

#### TGF-β

IFN-γ also activates the vitamin D-antimicrobial pathway, inducing antimicrobial peptide (e.g., cathelicidin) production, phagosome maturation, and autophagy (78). Importantly, IFN-γ and downstream vitamin D-dependent antimicrobial genes are preferentially expressed in TT and T1R skin lesions (79–81). Furthermore, the vitamin D-antimicrobial pathway is mediated via the vitamin D receptor (VDR), expressed by macrophages in response to TLR1 and TLR2 stimulation (82). Genotyping analysis identified an association of two functional *VDR* polymorphisms with leprosy phenotypes, including a missense M1T polymorphism (rs2228570; also known as FokI) of a *VDR* isoform associated with T1R (83). In addition, serum vitamin D3 levels and *VDR* mRNA expression correlate with the complexity and severity of LRs (84). Activated macrophages also produce the multifunctional cytokine TGF-β, and high levels of TGF-β have been identified in T1R biopsies (71). TGF-β and TNF-α can act synergistically to cause detachment and lysis of SCs, potentially contributing to SC killing and peripheral nerve damage in T1R (85). Together, these studies demonstrate the potential of pro-inflammatory cytokines as candidate biomarkers for leprosy phenotypes; however, more studies are needed.

### Pro-inflammatory Chemokines, Enzymes, and Growth Factors

C-X-C motif chemokine ligand 10 (CXCL10; also known as IP-10) is a pro-inflammatory chemokine that promotes T cell chemotaxis to sites of tissue inflammation (86). CXCL10 is produced by macrophages, T cells, and keratinocytes upon stimulation by IFN-γ. CXCL10 mRNA levels in the skin and protein levels in serum are elevated during T1R compared to before T1R (87). In addition, circulating CXCL10 levels decrease following treatment (88, 89). The CC chemokines, such as “regulated upon activation, normal T cell expressed and secreted” (RANTES; also known as CCL5) and monocyte chemoattractant protein-1 (MCP-1), recruit monocytes and lymphocytes to the lesion. Expression of both RANTES and MCP-1 is elevated in the skin lesions of T1R compared to non-reactional leprosy (90), suggesting a role of these chemokines in the activation of monocytes and T cells in T1R lesions.

Cyclooxygenase-2 (COX-2) is overexpressed during inflammation, and COX-2 expression is regulated by growth factors and cytokines, such as IL-1β, IL-6, and TNF-α (91). In skin biopsies from leprosy patients, foamy macrophages express COX-2, and expression is significantly higher in LL compared to TL (92). In addition, in T1R lesions, micro-vessels, nerve bundles, and isolated nerve fibers express COX-2, as well as vascular endothelial growth factor (VEGF) (93). VEGF and the endothelial cell receptor KDR (also known as VEGFR-2) are also overexpressed by granuloma cells, vascular endothelium, and the overlying epidermis in T1R (94). VEGF enhances prostaglandin (PG) production through COX-2 stimulation and PG synthase expression, causing vascular changes leading to tissue edema characteristic of T1R and potential nerve damage (93). Selective COX-2 inhibitors are currently used in several inflammatory conditions (95), and may be considered for T1R treatment to reduce acute symptoms and prevent long-term nerve damage (93). Th17 cells and γδ T cells.

Th17 cells are a distinct lineage of Th cells that play an important role in protection against intracellular pathogens (96). Human naïve CD4+ T cells differentiate into Th17 cells following exposure to IL-6, IL-1β, TGF-β, and IL-23 (46). Activated Th17 cells secrete the cytokines IL-17A, IL-17F, and IL-22, which induce epithelial cell production of IL-6, IL-1β, CXCL2, and CXCL8, attracting and activating inflammatory cells at the site of infection (46). The correlation of Th17 responses with the clinical forms of leprosy is similar to that of Th1 cells, indicating a role of Th17 cells in an effective immune response against *M. leprae* (9, 97, 98). *M. leprae* antigen-stimulated peripheral blood mononuclear cells (PBMCs) from patients with T1R and ENL showed significantly higher mRNA levels of *IL17A, IL17F, IL23, IL6*, and *IL21* than those derived from patients with TT and LL (99). *M. leprae*-stimulated PBMCs from patients with LRs exhibited a significantly higher frequency of CD4+IL-17+ T cells compared to those from non-reactional patients (99). Within granulomas, IL-17A and TGF-β are also abundant in biopsies from patients with T1R and ENL compared to those from patients with TT and LL (99). IL-17F also increases upon the development of T1R (97).

The association of Th17 cells with PB leprosy and increased Th17 activity during LRs suggest that patients with a greater frequency of Th17 cells acquire resistance to *M. leprae*. Moreover, due to reciprocal development pathways for Th17 cells and anti-inflammatory Tregs (100), decreased numbers of Tregs in favor of Th17 cells may be a plausible mechanism for LR development. Th17 cells may also contribute to host defense against leprosy by secretion of the antimicrobial cytokine IL-26 (101, 102). *IL-26* mRNA levels are higher in TT and T1R lesions compared to LL lesions, and IL-26 colocalizes to the greatest extent with CD4+ T cells, presumably Th17 cells (101).

γδ T cells are also a main source of IL-17 and IFN-γ in many diseases (103). γδ T cells composed 25–35% of the CD3+ T cells within granulomatous skin lesions of patients with T1R compared to just 5% in lesions of patients with other forms of leprosy (104). More recently, γδ T cells were demonstrated to be significantly enriched in the peripheral blood of patients with T1R and ENL compared to those with TT and LL (105).

### Anti-inflammatory Factors

Levels of the anti-inflammatory cytokine IL-10 are higher in patients with LL, consistent with hyporesponsiveness (106–108). Conversely, a reduction in the relative levels of IL-10 may correlate with the conversion of unresponsive T cells in LL/BL patients to activated pro-inflammatory T cells in LRs (107). In a BT-like murine model of leprosy, IL-10 suppression significantly augmented *M-leprae*-specific CD4+ and CD8+ T cell infiltration and permitted CD4+ T cells to penetrate and fragment nerve tissue (109). Furthermore, IL-10-production by *M. leprae*-stimulated PBMCs is reduced at the onset of T1R (110), suggesting that the breakdown of IL-10-mediated tolerance may be a general mechanism for T1R. Another study showed reduced expression of Treg-associated genes (*FOXP3, LAG3*) and IL-10 during the onset of T1R (110). It is hypothesized that the ratio of pro-inflammatory cytokines, such as IFN-γ, TNF-α, CXCL-10, IL-6, and IL-17, to anti-inflammatory cytokines, such as IL-10, may provide early and more accurate indicators for T1R rather than the absolute cytokine levels (88, 89, 110).

### Acute-Phase Proteins and Cortisol

APPs are a highly conserved class of proteins that play an essential role in the innate immune response by marking a pathogen for phagocytosis, a process called opsonization (111). APPs are secreted primarily by hepatocytes stimulated with TNF-α and IL-6 during the acute phase reaction/response, characterized by fever and activation of peripheral leukocytes, especially neutrophils (112). The most prominent APPs include C-reactive protein (CRP), which is used as a biomarker for inflammation, mannose-binding lectin (MBL), which activates complement by the lectin pathway, the coagulation factor fibrinogen, and the apolipoprotein serum amyloid protein A (SAA) (113). In addition to APPs, components of the complement system (e.g., C3b, C4b, and iC3b) and immunoglobulins opsonize molecules to promote phagocytosis (114). Notably, terminal complement complex (TCC) and iC3b of the complement system are valuable for the stratification of leprosy patients with or without T1R (115).

The stress hormone cortisol, which increases blood sugar levels and suppresses Th1-mediated immune responses (116), is elevated in T1R patients (117). In the skin, cortisol concentration is regulated by a reversible enzyme shuttle that deactivates cortisol by converting it to cortisone and *vice versa* (118). The activity of this enzyme shuttle is regulated by numerous factors, including cytokines (118). One potential mechanism for the development of LRs is a breakdown of the cortisol-cortisone enzyme shuttle, resulting in large fluctuations in cortisol concentration at the site of inflammation that requires exogenous steroids to regain balance (118). Thus, the cortisol-cortisone enzyme shuttle may be a biomarker for T1R and be useful for treatment customization. Consistent with this hypothesis, prednisolone treatment downregulates the expression of the gene encoding 11β-hydroxysteroid dehydrogenase type 2 (*HSD11B2*), which deactivates cortisol to cortisone, in the skin lesions of patients with T1R (119).

### Lipid Metabolites and Related Genes

A characteristic feature of LL is the accumulation of lipid droplets within *M. leprae*-infected macrophages, resulting in a foamy or xanthomatous appearance (120, 121). *M. leprae* infection induces lipid droplet formation by modifying the expression of host genes responsible for lipid metabolism, such as adipose differentiation-related protein (*ADRP*) and perilipin and hormone-sensitive lipase (*HSL*) (121–123). Specifically, *M. leprae* infection upregulates *ADRP* and downregulates *HSL* expression, suggesting *ADRP* and *HSL* as potential biomarkers for LRs (123).

There is close crosstalk between inflammatory and immune pathways and lipid mediators derived from polyunsaturated fatty acids, such as the omega-6 fatty acid arachidonic acid (AA) and the omega-3 fatty acids eicosapentaenoic acid and docosahexaenoic acid (124). Omega-3 and omega-6 fatty acid-derived lipid mediators are involved in regulating *M. leprae*-specific inflammatory and immune responses (125). AA is primarily metabolized into pro-inflammatory lipid mediators, such as 2-series PG, thromboxane, and 4-series leukotrienes, by cyclooxygenases and lipoxygenases (124). In contrast, some omega-3 and omega-6 fatty acids synthesize lipid mediators with anti-inflammatory and pro-resolution functions, including lipoxins, resolvins, protectins, and maresins (126, 127). Current evidence suggests that specialized pro-resolving lipid mediators (SPMs) are involved in the down-regulation of the innate and adaptive immune responses against *M. leprae* and that alteration in the homeostasis of pro-inflammatory lipid mediators and SPMs is associated with dramatic shifts in leprosy pathogenesis (128).

Serum metabolomic studies of patients with LRs identified 40 perturbed metabolites in T1R, with 71 dysregulated metabolites mapping to inflammatory lipid mediator pathways (98). Leukotriene B_4_ (LTB4) is released during the acute-phase response, inducing the recruitment and activation of neutrophils, monocytes, and other leukocytes at the site of inflammation and pro-inflammatory cytokine production (129). Consistent with the severe local inflammatory response in T1R, LTB4 levels are significantly higher in T1R compared with non-T1R patients (98, 124). As Th17 cells express LTB4 and its receptors, it is speculated that higher levels of LTB4 in T1R are due to the migration of Th17 cells (125, 130). PGD2 also acts as a pro-inflammatory mediator, regulating events such as Th2 cytokine production and leukocyte migration, and is present at higher levels in T1R compared with non-T1R patients (125).

PGE2 is an eicosanoid that causes vasodilation, attracts immune cells, and induces IL-10 synthesis; thus, PGE4 may limit non-specific inflammatory damage, favoring *M. leprae* persistence in MB patients through downregulation of macrophage functions (131). Consistent with an anti-inflammatory role of PGE2, non-T1R patients exhibit higher levels of PGE2 compared with T1R patients (98, 125). Reduced PGE2 levels in T1R indicate enhanced Th1 immune responses (125, 128).

Lipoxin A_4_ (LXA4) is neuroprotective, and higher levels of LXA4 are observed in non-reactional leprosy patients, suggesting that LXA4 preserves nerve function in leprosy (125). Similarly, resolvin D1 (RvD1) is an anti-inflammatory lipid mediator that suppresses the synthesis of LTB4 while favoring LXA4 synthesis. Resolvins also inhibit neutrophil infiltration, support an M2 macrophage phenotype switch, enhance bacterial phagocytosis, induce Treg differentiation and consequent IL-10 production, and inhibit Th1 and Th17 cell functions (132). Higher levels of RvD1 are observed in non-T1R patients compared with those developing T1R (98, 125). As LXA4 and RvD1 are predominant in non-reactional leprosy patients, it is speculated that they play a role in the maintenance of the disease, avoiding exacerbated inflammatory responses, which could be deleterious for both the pathogen and host (98, 125). Consistent with this hypothesis, patients with high bacterial load (e.g., LL and BL patients) exhibit the highest levels of LXA4 and RvD1 (98, 125). Together, these findings suggest that alterations in the homeostasis of pro-inflammatory lipid mediators and SPMs could cause the Th1-mediated pathology observed in T1R (128).

Similar to serum metabolites, urine metabolites are easily accessible from a non-invasive body fluid. Exploratory metabolomic analysis of a prospective cohort of Nepalese leprosy patients with and without LRs showed that cross-sectional urinary metabolic signatures at the time of T1R diagnosis distinctly differed from those before LRs (133). Thus, urine metabolites may also predict the onset of LRs. While the above-mentioned correlates are frequently reported in TIR, none of them has been firmly established as a reliable biomarker to be able to diagnose T1R on its own.

### Host Transcriptomes

Host transcriptomic biomarkers reflect actively ongoing immune responses and may be used to profile LRs. Transcriptomic analysis of skin tissue, whole blood, and PBMCs of leprosy patients has identified several differentially expressed genes characteristic of LRs (110, 134, 135). In agreement with a Th1 pro-inflammatory cytokine profile, expression of pro-inflammatory cytokine genes was up-regulated in independent studies assessing mRNA expression in whole blood and *M. leprae*-stimulated PBMCs (110, 134, 135). Monitoring whole blood transcriptomics of a leprosy patient before, at onset, and after T1R treatment revealed that IFN-inducible transcripts, *VEGF*, and CTL response-associated genes, including granulysin, perforin, and granzymes A and B, were up-regulated during T1R (110). In contrast, Treg-associated genes were down-regulated, and there was only minimal detection of IL-4 and IL-13 (110). A unique 44 gene signature, including genes associated with AA metabolism, was identified in *M. leprae* antigen-stimulated PBMCs from patients with T1R (135) and both T1R and ENL exhibited increased gene expression of C1q (134).

### *M. leprae* and Its Derivatives

In addition to host-derived biomarkers, a few studies have investigated the components or derivatives of *M. leprae* for leprosy diagnosis and monitoring of treatment efficacy (136–139). Specifically, *M. leprae* antigenic determinants have been demonstrated in dermal macrophages and SCs during T1R (38). A study of patients with slit-skin smear negative, single lesion, PB leprosy demonstrated an association between T1R and the presence of *M. leprae* DNA in skin lesions (138). In particular, expression of the *M. leprae*-specific genes *accA3* and *hsp18* was higher in biopsies from T1R patients compared with those from non-reactional leprosy patients (136, 140). Interestingly, *M. leprae* genome displays gene decay significantly and contain large numbers of pseudogenes and non-coding regions (141). Our lab has shown that RNA transcripts are generated from *M. leprae* pseudogenes and non-coding regions (52, 120, 142), and these transcripts could be a valuable biomarker for the disease phenotype (139).

## Pathogenesis and Related Biological Agents as Biomarkers for ENL

ENL primarily affects individuals with BL and LL leprosy but may also occur in a small percentage of individuals with BB leprosy (143). Approximately 10% of patients with BL leprosy and up to 50% of those with LL leprosy will develop ENL (143). The risk for ENL in patients with BL leprosy and a bacteriological index (BI) ≥4 is 5.2 times greater than patients with BL leprosy and a BI <4 (144). ENL may share some disease mechanisms with T1R; however, ENL pathogenesis appears much more complex, and the underlying mechanisms for ENL remain unclear.

### Immune Complexes

A longstanding theory is that immune complexes and type III hypersensitivity reactions are involved in ENL pathogenesis (145). Type III hypersensitivity occurs following inadequate clearance of deposited immune complexes (also known as antigen-antibody complexes), leading to an inflammatory response and attraction of leukocytes (145). Consistent with this theory, skin biopsies of ENL patients show deposition of complement proteins and immunoglobulins in the dermis, similar to an Arthus reaction (146). In addition, patients with active ENL exhibit lower circulating C1q protein levels, but higher C1q gene expression in both skin lesions and peripheral blood, compared with non-reactional LL patients (134, 147), suggesting the consumption of C1q in the formation of immune complexes. Therefore, circulating C1q has potential as a diagnostic biomarker for ENL. A study of 109 non-related leprosy patients in Brazil reported an increased risk of ENL in patients with a deficiency in the complement protein C4B (C4B^*^Q0), potentially leading to abnormal immune responses due to inadequate immune complex clearance (148). Moreover, circulating immune complexes against phenolic glycolipid-1 (PGL-1) and major cytosolic proteins of *M. leprae* are found in patients with ENL (149). Although these studies show the presence of immune complexes in ENL, this may be an epiphenomenon, and the causative role of immune complexes in ENL pathogenesis remains unclear (15).

### Neutrophils

Neutrophils contribute to the early phases of leprosy pathogenesis by phagocytosing *M. leprae* and releasing pro-inflammatory mediators and are considered a histological hallmark of ENL (150). A study of ENL patients showed that neutrophils composed 30% of skin biopsies within 72 h after ENL onset but composed only 1.6% after 9–12 days (150). Higher neutrophil-to-lymphocyte ratios (NLRs) are significantly associated with systemic inflammation and reflect non-specific acute inflammatory responses mediated by neutrophils (151). In a study of 123 patients with leprosy, including 56 with T1R and 42 with ENL, patients with ENL had the highest NLR, and the NLR had a sensitivity of 81% and specificity of 74% for ENL diagnosis (134).

It has recently been shown that PGL-I interacts with complement receptor 3 (CR3) on macrophages, polymorphonuclear neutrophils and dendritic cells (152). This binding of CR3 by PGL-I triggers Syk tyrosine kinase, inducing calcineurin-dependent nuclear translocation of the transcription factor NFATc, eventually rewiring host cytokine responses in leprosy (152). PGL-I that triggers this pathway upon CR3 binding sustains IL-1β production by macrophages, IL-10 by polymorphonuclear neutrophils, and IL-2 by dendritic cells, which coordinately regulates neutrophils infiltration in ENL patients (152).

Endothelial cell expression of the leukocyte-endothelial cell adhesion molecule E-selectin is promoted by IL-1β and IFN-γ following activation of TLR2 and Fc receptors, allowing for neutrophil adherence and migration to sites of inflammation (153, 154). E-selectin is expressed in a vascular pattern, and expression is highest in ENL skin lesions compared to non-reactional LL leprosy (153). Furthermore, transcriptomic analysis of leprosy skin lesions identified ENL-specific neutrophil and endothelial cell gene networks involved in vasculitis associated with tissue injury (155). Consistent with a role of this pathway in ENL, the effective ENL treatment thalidomide inhibits E-selectin-mediated neutrophil recruitment (153).

Resting neutrophils express low levels of the cell surface receptor CD64 (FcγRI); however, stimulation by gram-negative bacteria increases expression (156). CD64 is also an early biomarker and predictor of severity for ENL (157, 158). Circulating and *in situ* neutrophils in ENL, but not non-reactional leprosy, express CD64 (157, 158). CD64 upregulation in ENL may occur due to the release of fragmented components of *M. leprae* after initiating MDT (157, 158). Neutrophils produce the majority of TNF-α and IL-8 associated with tissue damage in ENL (159), consistent with the role of CD64 in the upregulation of pro-inflammatory cytokine production (157, 158). Thalidomide suppresses neutrophil TNF-α secretion, suggesting another mode of action for this treatment (159).

Neutrophil IL-10 receptor 1 (IL-10R1) was recently proposed as a potential biomarker and target for ENL treatment (160). A recent study found that in contrast to neutrophils from non-reactional leprosy patients, a subpopulation of neutrophils in the circulation and skin lesions of ENL patients exclusively expressed IL-10R1, enabling response to IL-10 (160). IL-10R1 expression on ENL neutrophils was further increased during thalidomide treatment (160). In addition, neutrophils from ENL but not non-reactional leprosy patients secreted detectable levels of inflammatory cytokines *ex vivo*, which was blocked by the addition of IL-10 (160). Expression of IL-10R1 by ENL neutrophils may reflect a compensatory mechanism to regulate inflammation during ENL; however, the causative role of neutrophils in ENL has yet to be determined (15).

### T Cells

Some studies showed that ENL, like T1R, is induced primarily by a T cell-mediated immune response (15, 161). CD8+ clones derived from LL lesions secrete large amounts of IL-4 and minimal IFN-γ (162, 163). In response to *M. leprae* or *M. leprae* antigens, lesion-derived CD8+ T cells do not proliferate and limit the proliferation and cytokine secretion of bystander T cells (162, 163). Thus, CD8+ T cells may induce *M. leprae*-specific T cell anergy (162, 163). In contrast, TT skin lesions display a predominance of CD4+ T cells that secrete high amounts of IFN-γ (164, 165). Likewise, there is an increase in CD4+ T cells and a decrease in CD8+ T cells in both the skin and blood of patients with ENL compared with non-reactional LL patients (166, 167), supporting the involvement of T cells in ENL. However, these early studies need to be re-evaluated in the context of CD4+CD25+ Tregs.

Similar to T1R, Th17 and γδ T cells are significantly enriched in the peripheral blood of patients with ENL compared with non-reactional leprosy patients (99, 105) (see section 2.5), suggesting a role for these T cells in ENL. In addition, studies reported reduced Treg levels in circulation and *in situ* in ENL but not T1R (106, 168). As Tregs suppress Th1 cells, the reduction of Tregs may explain the higher proportion of effector T cells in ENL (169).

### Cytokines, Chemokines, and Enzymes

Although the clinical presentations of T1R and ENL are distinct, they share similar pro-inflammatory cytokine profiles during disease progression. In ENL, enhanced Th1 responses to *M. leprae* and macrophage activation are reflected by elevated expression of IFN-γ, TNF-α, IL-1β, IL-2, and IL-6 in the affected tissues and serum (170–172). Most studies found that high TNF-α serum levels correlated with ENL, and levels decreased significantly during thalidomide treatment (173). Consistent with this finding, the primary mechanism of action of thalidomide is TNF-α suppression, although other mechanisms may apply (174). The chimeric anti-TNF-α monoclonal antibody infliximab is also effective for the treatment of ENL, further supporting an important role of TNF-α in ENL pathogenesis (175). High serum IFN-γ levels also correlate with ENL (173), and intradermal injection of IFN-γ is associated with an increased frequency of ENL (176). Furthermore, although thalidomide reduces ENL frequency, it also eliminates IFN-γ-mediated bacillary killing (176). Most studies suggest a prognostic role of IL-1β for ENL (65, 177). IL-6 promotes cell-mediated immune reactions, notably by stimulating IL-17 and inhibiting Tregs (68). A study found independent associations of two IL-6 polymorphisms, rs1800795 and rs2069840, with ENL (178), which influence *IL6* expression and correlate with circulating IL-6 levels, respectively (179). Thus, IL-6 is also implicated in ENL pathogenesis and is a potential predictive biomarker for ENL. IL-17 increases upon ENL onset and thalidomide suppresses Th17 responses (9, 47), supporting a role of Th17 cells in the immunopathogenesis of ENL.

In contrast to T1R, a predominant Th2 cytokine profile has been observed in ENL with increased expression of IL-6, IL-8, and IL-10 and sustained production of the Th2 cytokines IL-4 and IL-5 (180), indicating a role of humoral immunity in ENL. In a cohort of 6 cases each of T1R and ENL, increased expression of IL-10 was observed in ENL, but not T1R (65). IL-7 is a key regulator of B cell development and proliferation and is essential for the survival of naïve and memory T cells, especially CD4+ memory cells (181, 182). Elevated circulating IL-7 levels were detected in ENL (67), implicating a role for both B cell- and T cell-mediated immunity in ENL.

C-C motif chemokine ligand 11 (CCL11), a chemokine produced by monocytes, has also been identified as a potential plasma marker of ENL (67). CCL11 is a potent chemoattractant for eosinophils and Th2 lymphocytes (183). Global transcriptional profiles of PBMCs also revealed CCL2, CCL3, and CCL5 as potential biomarkers for ENL (134). Matrix metalloproteinases (MMPs) are a family of proteolytic enzymes responsible for extracellular matrix (ECM) remodeling and regulation of leukocyte trans-ECM migration, an important step in inflammatory processes as well as infectious diseases (184). MMPs are produced by skin cells, such as keratinocytes, Langerhans cells, and dermal fibroblasts (184). Serum MMP-9 levels are elevated in patients with LRs, and *MMP* mRNA levels are higher in skin biopsies of patients with LRs, especially in ENL, and correlated with skin biopsy IFN-γ and TNF-α levels (185).

### Humoral Immunity

Antibodies (Abs) against *M. leprae* are the main players in the humoral immune response involved in leprosy pathogenesis. The role of humoral immune responses in immune defense against intracellular pathogens such as *M. leprae* is generally thought to be irrelevant. Instead, Ab production at the site of infection may contribute to the immunopathology and tissue injury observed in leprosy, as ENL pathogenesis has been attributed to Abs and immune complex deposition (see section 3.1). ENL patients exhibit elevated IgG1-secreting B cells (186), with lower concentrations of *M. leprae*-specific IgG1 and IgG3 (187). As LRs are initiated by *M. leprae* antigens and bacterial load is associated with anti-*M. leprae* Ab levels (188), Ab levels at the time of leprosy diagnosis have been evaluated as predictive biomarkers of LRs. High levels of anti-PGL-1 Abs at diagnosis or after treatment have been associated with a higher risk of developing LRs, especially ENL (189). MB patients who subsequently developed ENL had increased levels of IgM, IgG1, and C3d before ENL onset, suggesting that they are potential biomarkers for ENL (190).

Leprosy Infectious Disease Research Institute Diagnostic-1 (LID-1) is a fusion protein of ML2331 and ML0405 recognized by *M. leprae*-specific Abs, and persistently high levels of anti-LID-1 Abs might be a useful tool to predict ENL (188, 191, 192). In addition, in a study of 452 non-reactional leprosy patients at diagnosis, baseline serum anti-LID-1 Ab levels were elevated in patients with a high BI and predicted the development of ENL with a sensitivity of 71% and specificity of 80% (191). In another study, serum anti-LID-1 Ab levels were associated with LRs as well as neuritis of leprosy (192). Furthermore, elevated levels of Abs against LAM, a polysaccharide antigen present in *M. leprae*, are associated with the development of T1R (193).

Negera et al. demonstrated an increase in activated memory B cells in untreated patients with ENL, suggesting a role of memory B cells in the pathology of ENL (194). Untreated ENL patients also exhibited a reduction in the number of tissue-like memory B cells (TLM) compared to LL patients (194). Furthermore, the study found that the percentage of total B cells in peripheral blood was not significantly different between patients with LL and ENL; however, treatment significantly reduced the proportion of B cells from 9.5% to 5.7% in patients with ENL, suggesting that the depletion of B cells could be an effective treatment for ENL (194).

### APPs and Procoagulant Factors

APPs have also been proposed as potential biomarkers for ENL. Serum pentraxin-3 (PTX-3) levels are higher in MB patients before the onset of acute ENL, persist during LR, and are reduced by thalidomide (195). PTX-3 binds with high affinity to the complement component C1q, which could explain why C1q levels in the circulation are inversely correlated with ENL progression (134, 147). CD64 expression on neutrophils correlates positively with PTX-3 serum levels in ENL, suggesting that CD64/PTX-3 exacerbates inflammation in ENL patients (195). PTX-3 also colocalizes with the neutrophilic marker myeloperoxidase (MPO) in ENL lesions, and the high expression of PTX-3 in ENL could result from high neutrophil numbers (195).

SAA and CRP, systemic markers of inflammation, are elevated in ENL, indicating active inflammatory responses (196). In addition, serum concentrations of TNF-α and CRP are positively correlated (197). At high concentrations, CRP can enhance the acute inflammatory process in ENL, favoring increased macrophage activation and phagocytosis, contributing to the elimination of damaged cells and bacilli, and modulating the proportion of T cell subsets (197). Using serum proteome analysis with two-dimensional gel electrophoresis and mass spectrometry, another acute-phase protein, α-acid glycoprotein (AGP), was found to be increased in the serum of untreated ENL patients (198). AGP levels decreased to normal levels after treatment with MDT and thalidomide (198). Furthermore, an LL patient who progressed into ENL exhibited a stage-dependent increase in AGP, supporting the use of AGP levels as a biomarker for ENL (198). Serum proteome analysis of patients with ENL also showed a significant increase in an isoform of the haptoglobin α2 chain compared with non-reactional leprosy patients (199).

Hemostatic disorders are frequently associated with acute and chronic infections, as exemplified by platelet functions, blood coagulation, and fibrinolysis, and are intimately correlated with immune responses (200). Leprosy patients can develop hemostatic abnormalities, such as atypical lipid clot mass formation during serum harvesting, deep thrombophlebitis, and pulmonary embolism (201). Patients with ENL have prolonged activated partial thromboplastin times, high fibrinogen and platelet titers, and platelet activation (202, 203). Procoagulant profiles of 40 reactional and non-reactional MB leprosy patients identified components of neutral lipids in the leprosum clot highly enriched in fibrin, inter-α-trypsin inhibitor family heavy chain-related protein (IHRP), and the complement components C3 and C4 (204). Among these components, plasma fibrinogen levels were increased in patients developing ENL; thus, demonstrating its potential as a predictive biomarker of ENL (204).

### Host Genetics

Several host genetic polymorphisms have also been identified as risk or protective factors for ENL. In a study in Bangladesh, a non-synonymous polymorphism of *TLR1*, rs4833095, which causes a substitution of asparagine to serine (N248S) in the external recognition site, was identified as protective against ENL (205). MBL is involved in pathogen recognition and clearance by the innate immune response (206). MBL activates the complement pathway by co-opting MBL-associated serine proteases (MASPs) (207), cleaving the complement proteins C2 and C4 and inducing opsonization (207). Alleles of the *C4B* gene are also associated with LL and ENL susceptibility (148). Natural resistance-associated macrophage protein 1 (NRAMP1) mediates the transportation of divalent metals (208), and an exon 3'UTR SNP 274C/T in its encoding gene (*SLC11A1*) is associated with LRs. In a Brazilian study, the presence of the “C” allele on this SNP was a risk factor for T1R and protective against ENL (209). Nucleotide-binding oligomerization domain-containing protein 2 (NOD-2) recognizes bacterial molecules and stimulates an immune response (210). SNPs in the *NOD-2* gene are strongly associated with LRs (211). Together, these findings support a role for innate immunity in ENL pathogenesis.

## Prospective

Potential biomarkers, including genetic, serological, metabolomic, and transcriptomic correlates, for LRs (Table 1) have been continuously proposed as scientists unravel the mystery of LR pathogenesis, which appears to be jointly determined by pro- and anti-inflammatory host immune responses. However, conflicting data among studies are common, possibly due to the low number of new leprosy cases, inappropriate controls, significant patient heterogeneity, inconsistent sampling, and various research methods. Therefore, future studies may better focus on biomarker signatures that contain multiple correlates (e.g., the ratio between pro- and anti-inflammatory cytokines, gene transcripts, and cell numbers). Such biomarker signatures should be more reliable than monitoring the absolute levels of a single correlate. For establishment of host genetic biomarkers, genotype profiling and relevant GWAS data may be used to calculate the polygenic risk score to estimate a patient's liability to LRs. Also, interdisciplinary research of medicine, bioinformatics, and mathematics would merit the construction of tangible algorithms for referring those proposed biomarkers, which should be as practical as possible. Furthermore, for serum and tissue biomarkers, future studies should better compare both inter-individual and intra-individual longitudinal levels, as some biomarkers may vary considerably inter-individually, and the latter may be useful to monitor and customize treatments. As many studies suffered from low number of new leprosy cases, multi-national cooperation and multi-center joint research is needed to overcome the limitation. Finally, since areas hyperendemic for leprosy are mostly seen in less-developed countries, the development of biomarkers suitable for field-friendly diagnostic tools and telemedicine should be prioritized, which would require collaborative development of medicine, engineering materials science, and communication engineering. In near future, cell phone applications using artificial intelligence to help recognize leprosy and LRs skin based on images uploaded by patients may even become available.

**Table 1:** Correlates in T1R and ENL.

**Correlates**	**T1R**	**ENL**
**Host genetic correlates**		
*TLR1* (57, 205)	+	+
*TLR2* (53–55)	+	
*TLR4* (54, 56)	+	
*TNFSF15-TNFSF8* (72, 73)	+	
*LRRK2* (74)	+	
*VDR* (83)	+	+
*IL-6* (178)		+
*C4B* (148)		+
*NRAMP-1* (209)	+	+
*NOD-2* (211)	+	+
**Circulating proteomic correlates**		
**TNF-α** (65, 173)	+	+
**IFN-γ** (65, 173)	+	+
**IL-1β** (65, 177)	+	+
**IL-6** (67)	+	+
neopterin (77)	+	
**CXCL-10/IP** (67, 87–89)	+	
γδ T cells ratio (105)	+	+
IL-10 (65, 110)	+	+
CRP (117, 196, 197)	+	+
iC3b (115)	+	
LTB4 (98, 124)	+	
PGD2 (125)	+	
PGE2 (98, 125)	+	
RvD1 (98, 125)	+	
C1q (134, 147)	+	+
Immune complex against PGL-1 and MCP (15)		+
NLR (212)		+
CD64/FcγRI (157, 158)		+
IL-10R1 (160)		+
CD4: CD8 ratio (166, 167)		+
IL-7 (67)		+
CCL-11 (67)		+
Anti-LAM (193)	+	
anti-LID-1 (188, 191, 192)		+
PTX-3 (195)		+
SAA (196)		+
AGP (198)		+
Fibrinogen (204)		+
**Proteomic correlates in skin and nerve tissues**		
**TNF-α** (69–71, 185)	+	+
**iNOS** (63)	+	+
**TGF-β** (71, 99)	+	+
**CXCL-10/IP** (87)	+	
CCL-5/RANTES and MCP-1 (90)	+	
COX-2 (93)	+	
VEGF and KDR (94)	+	
IL-17A (99)	+	+
IL-17F (97)	+	
γδ T cells ratio (104)	+	
11β-HSD2 (119)	+	
e-selectin (153)		+
CD64/FcγRI (157, 158)		+
IL-10R1 (160)		+
CD4:CD8 ratio (166, 167)		+
MMP-9 (185)		+
**Host transcriptomic correlates**		
*IL-26* (101)	+	
*IL-10* (110)	+	
*VEGF* (110)	+	
IFN-inducible genes (*OAS1/2, GBP1/5, IFI44, IFI44L, IFIT5, IFIH1*) (110)	+	
Cytotoxic T cell response-associated genes (*GNLY, GZMA/B, PRF1*) (110)	+	
Treg-associated genes (*FOXP3, LAG3*) (110)	+	
A 44 gene signature (135)	+	
*C1q* (134)	+	+
*CCL-2, CCL-3*, and *CCL-5* (134)		+
**Urinary metabolic signatures** (133)	+	

*Correlates found in T1R and ENL, including genetic, serological, metabolomic, and transcriptomic factors. (): reference No.; +: implicated. Comparative more important correlates are bolded*.

## Author Contributions

YL drafted the manuscript. MK, KT, AK, YN, NI, and KS critically revised the manuscript. All authors contributed to the article and approved the submitted version.

## Funding

This work was supported by National Natural Science Foundation of China (81900712).

## Conflict of Interest

The authors declare that the research was conducted in the absence of any commercial or financial relationships that could be construed as a potential conflict of interest.

## Publisher's Note

All claims expressed in this article are solely those of the authors and do not necessarily represent those of their affiliated organizations, or those of the publisher, the editors and the reviewers. Any product that may be evaluated in this article, or claim that may be made by its manufacturer, is not guaranteed or endorsed by the publisher.
